# Bronchopulmonary Dysplasia: Pathogenesis and Pathophysiology

**DOI:** 10.3390/jcm12134207

**Published:** 2023-06-22

**Authors:** Nilesh Dankhara, Ira Holla, Sumana Ramarao, Renjithkumar Kalikkot Thekkeveedu

**Affiliations:** Department of Pediatrics, University of Mississippi Medical Center, Jackson, MS 39216, USA; iholla@umc.edu (I.H.); sramarao@umc.edu (S.R.); rkalikkot@umc.edu (R.K.T.)

**Keywords:** bronchopulmonary dysplasia, sepsis, mechanical ventilation, pathogenesis, inflammation, PDA

## Abstract

Bronchopulmonary dysplasia (BPD), also known as chronic lung disease, is the most common respiratory morbidity in preterm infants. “Old” or “classic” BPD, as per the original description, is less common now. “New BPD”, which presents with distinct clinical and pathological features, is more frequently observed in the current era of advanced neonatal care, where extremely premature infants are surviving because of medical advancements. The pathogenesis of BPD is complex and multifactorial and involves both genetic and environmental factors. This review provides an overview of the pathology of BPD and discusses the influence of several prenatal and postnatal factors on its pathogenesis, such as maternal factors, genetic susceptibility, ventilator-associated lung injury, oxygen toxicity, sepsis, patent ductus arteriosus (PDA), and nutritional deficiencies. This in-depth review draws on existing literature to explore these factors and their potential contribution to the development of BPD.

## 1. Introduction

Bronchopulmonary dysplasia (BPD) is a chronic lung disease that affects premature infants. It is the most common respiratory morbidity in preterm infants who require mechanical ventilation and/or supplemental oxygen therapy. BPD is characterized by inflammation, injury to the developing lung, and impaired growth of the alveoli and blood vessels. BPD is a complex disorder with multiple potential etiologies contributing to its pathogenesis. Extreme prematurity, defined as gestational age less than 28 weeks, has been consistently shown to be the most important risk factor for BPD. Understanding the underlying pathology and pathogenesis of BPD is important for developing effective prevention and treatment strategies for this common respiratory morbidity in premature infants. Here, in this review, we attempted to provide a summary of the available literature on the pathology and pathogenesis of BPD.

## 2. Pathology

BPD was initially described in moderate and late preterm infants who experienced severe respiratory failure after initial respiratory distress syndrome, requiring high pressure and oxygen on mechanical ventilation. This is known as the “classic” or “old” BPD. Infants with this type exhibit severe lung injury, including necrotizing bronchiolitis, large airway injury, pulmonary arterial wall thickening, diffuse emphysema, pulmonary fibrosis, and cystic changes in the lung parenchyma [1].

Dr. Northway and colleagues described four histopathological stages of disease progression in the classic BPD. The first stage, which typically occurs within the first few days after birth, is characterized by diffuse patchy areas of atelectasis, hyperemia, lymphatic dilatation, necrosis of mucosa, and loss of ciliated cells. Hyaline membranes are also present. The second stage usually manifests in the next 4–10 days of life. It is characterized by a widespread area of necrosis and repair of alveolar epithelium, emphysematous coalescence of alveoli, and persistence of alveolar hyaline membrane and atelectasis of stage 1. The third and fourth stages are considered progressive and advancing lung disease. In the third stage, persistent areas of injury to alveolar epithelium and emphysematous changes resulting from alveolar coalescence with surrounding atelectasis persist, although hyaline membranes are significantly less. There is also interstitial edema, septal thickening, and excessive mucus secretion. The fourth stage, which occurs after a month, is the chronic disease phase, where there is an increased number and size of emphysematous areas, progressive fibrosis, and higher numbers of macrophages, foam cells, and histocytes. Additionally, there is an increased distance between alveolar epithelium and pulmonary capillaries, with tortuous lymphatics [1,2].

Over the past few decades, there have been significant advancements in neonatal care, such as the use of surfactants, gentle ventilation strategies, and antenatal steroids. These have greatly improved the survival rates of premature infants, including those born extremely premature [3]. However, despite these advancements, the incidence of BPD has not decreased. In fact, the number of infants surviving with BPD has increased because of the improved survival of extremely premature infants [1]. The current form of BPD seen in preterm babies in the post-surfactant era does not follow the stages described by Dr. Northway. The advances in neonatal intensive care practices, as mentioned above, have led to a milder form of the disease. The Eunice Kennedy Shriver National Institute of Child Health and Human Development/National Institutes of Health workshop on BPD has also highlighted the changing nature of the disease and the pattern of lung injury in surviving extremely premature infants.

The pathology of new BPD involves a complex interplay of factors, including delayed lung development and lung injury/repair. Ventilator-induced lung injury causes characteristic features of tissue injury, such as interstitial thickening and alveolar infiltration of inflammatory cells [4,5]. Endothelial and epithelial dysfunction and disruption of the alveolar–capillary barrier cause pulmonary edema and increased protein permeability. The diffuse alveolar damage in lung injury can be divided into three interrelated and overlapping phases” (i) the exudative phase of edema and hemorrhage; (ii) the proliferative phase of organization and repair; and (iii) the fibrotic phase or late resolving phase. The acute exudative phase is characterized by various features, including interstitial and intra-alveolar edema, hemorrhage, necrosis of type I pneumocytes and endothelial cells, and the formation of hyaline membranes with extensive thrombosis. In the organizing proliferative phase, hyaline membranes are incorporated into the alveolar septa, and there is parenchymal necrosis, chronic inflammation, type II pneumocyte hyperplasia, squamous metaplasia, and luminal organizing fibrosis. In the late resolving phase, granulation tissue is incorporated into the alveolar septa, leading to the hyalinization of alveolar walls, collagenous fibrosis, and mural fibrosis [5,6].

Compared to old BPD, lung pathology from infants dying from new BPD shows less fibrosis, and uniform inflation, with an absence of significant metaplasia of airway epithelial cells and underdeveloped pulmonary microvasculature. The alveoli in infants with new BPD are fewer in number and larger in size, which is known as alveolar simplification [7,8], and there is an increase in elastic tissue, but abnormal septation of alveoli leads to a reduction in surface area and abnormal development of pulmonary vasculature [7].

## 3. Pathogenesis of Bronchopulmonary Dysplasia

It is important to note that the pathogenesis of BPD is complex and multifactorial, and many of these risk factors likely interact with each other to contribute to the development of BPD. In this section, we describe some of the most common risk factors involved in the pathogenies of BPD.

### 3.1. Prenatal Risk Factors

#### 3.1.1. Maternal Smoking

Multiple studies have shown that in utero exposure to tobacco impairs respiratory function in preterm infants, otherwise healthy infants, and children [9,10]. The adverse effects of tobacco chemicals, particularly nicotine, on lung development have been observed in animal models [10]. Several studies have described in utero smoke exposure as an independent risk factor for the development of BPD [11,12]. Interestingly, a recent meta-analysis reported that the definition of BPD as oxygen requirement at different time points (postnatal day 28 vs. postmenstrual age 36 weeks) can impact the association between maternal smoking and BPD, with some studies not finding an association with maternal smoking when BPD is defined as oxygen requirement at postnatal day 28 but had significant association when defined as oxygen requirement at postmenstrual age 36 weeks [13].

Thus, promoting smoking cessation during pregnancy is crucial to protect the respiratory health of the mother and the developing fetus [10]. 

#### 3.1.2. Chorioamnionitis

Chorioamnionitis, an infection of the fetal membranes or placenta, has been a controversial factor in the development of BPD [14]. Animal studies have shown that chorioamnionitis initially supports lung maturation but eventually disrupts further lung development, resulting in BPD [15]. Human studies have also demonstrated an association between chorioamnionitis and BPD. A recent meta-analysis that included 158 studies confirmed chorioamnionitis as an important risk factor for all BPD vs. for moderate to severe BPD depending on how BPD was defined in those studies [16]. Another prospective cohort study of <32 week preterm infants with a histological diagnosis of chorioamnionitis with evidence of fetal inflammatory response syndrome also identified it as a significant risk factor for chronic lung disease [17]. However, there are conflicting findings as well. A large cohort study of VLBW infants over twenty-five years suggests that chorioamnionitis is not an independent risk factor for BPD but rather contributes to preterm delivery and neonatal sepsis, both of which are associated with BPD [18]. 

Ureaplasma colonization, an independent risk factor for preterm labor and chorioamnionitis, has also been implicated in the development of BPD. Animal studies have shown that Ureaplasma-induced lung inflammation mimics changes seen in BPD lung pathology, but the effects may vary among different animal species [19]. Human studies have shown that Ureaplasma colonization leads to inflammatory changes in preterm lung specimens [20]. A systematic review of studies defining BPD as oxygen requirement at different time points has concluded that Ureaplasma colonization is associated with BPD, but the most significant effects were reported only in small studies with concerns for bias [21].

In conclusion, the relationship between chorioamnionitis, Ureaplasma colonization, and BPD is not yet fully understood, and more research is needed to determine the mechanisms and implications of these associations. Because of the complexities of the studies, including variations in the study designs, definitions, and confounding factors, it is difficult to determine a clear causative link.

#### 3.1.3. Pregnancy-Induced Hypertension (PIH)

Maternal hypertensive disorders can cause preterm delivery and intrauterine growth restriction (IUGR), both of which are independent risk factors for BPD. PIH induces placental insufficiency, resulting in abnormal vasculature, decreased placental growth factors, suppressed vascular endothelial growth factors (VEGFs), and oxidative stress [22]. These pathophysiological changes may contribute to impaired lung development and subsequent development of BPD. However, despite extensive research, there is currently no conclusive evidence supporting this hypothesis. Studies investigating the association between PIH and BPD have yielded conflicting results [23]. A recent systematic review found no significant association between PIH and BPD in preterm infants born before 37 weeks [24]. Conversely, another meta-analysis of 211 studies involving 347,963 infants showed an increased risk of BPD in fetuses exposed to maternal hypertension accompanied by fetal growth restriction (FGR) but not with maternal hypertension alone [25].

In conclusion, while maternal hypertensive disorders are established risk factors for preterm delivery and IUGR, their direct association with BPD remains inconclusive. This highlights the need for more robust research, including well-designed prospective studies, to elucidate the role of maternal hypertensive disorders in the development of BPD in preterm infants.

#### 3.1.4. Intrauterine Growth Restriction (IUGR)

The risk of BPD is increased in preterm infants who are affected by fetal growth restriction (FGR) compared to infants who are appropriate for gestational age (AGA) [26]. Growth-restricted monozygotic twins have a higher risk of BPD, suggesting early effects of FGR on lung development contribute to the predisposition [27]. Preclinical studies in IUGR animal models have provided further insight into the impact of FGR on fetal lung development. These studies have shown that BPD in FGR is associated with decreased VEGF receptor activity in the lungs, exaggerated mitochondrial oxidative response, decreased pulmonary vascular and alveolar growth, abnormal pulmonary endothelial cell function, restricted expression of surfactant protein genes, and upregulation of transforming growth factor beta [28,29].

In conclusion, IUGR increases the risk of BPD in preterm infants, and multiple mechanisms have been suggested to explain this association. Further research is needed to develop targeted interventions for improving lung health in this vulnerable population.

#### 3.1.5. Genetics

The genetic factors contributing to the development of BPD are complex and not fully understood. Two independent twin studies have suggested a heritable component to BPD, with higher rates observed in monozygotic (i.e., identical) twins compared to dizygotic (i.e., nonidentical) twins [30,31]. However, there is no consensus on specific genes or genetic factors definitively associated with BPD. 

Recent genetic techniques, such as genome-wide association studies and whole exome sequencing, have identified some candidate genes that may be associated with BPD. These include genes encode VEGF, Toll-like receptors, tumor necrosis factor, interleukins, and matrix metalloproteinases [32,33,34,35]. Additionally, some studies have suggested that different biological pathways may contribute to the heritability of BPD depending on the severity of the condition [36]. For example, mild/moderate BPD may involve different genetic factors compared to severe BPD or death. This underscores the complexity of the genetic basis of BPD and highlights the need for further research to better understand the underlying mechanisms.

Multiple genes and genetic interactions may contribute to the development of BPD, and further research with rigorous methodologies, larger sample sizes, and diverse populations is needed to unravel the complex genetic architecture of this disorder.

### 3.2. Postnatal Risk Factors

#### 3.2.1. Mechanical Ventilation and Ventilator-Induced Lung Injury (VILI)

Mechanical ventilation and associated injuries are crucial risk factors in the development of BPD. The pathogenesis of VILI is multifactorial and results from complex interactions between the ventilator and patient-related factors. 

Ventilator-related factors, such as barotrauma (injury caused by excessive airway pressure), volutrauma (injury caused by excessive tidal volume), atelectrauma (injury caused by repetitive opening and closing of alveoli), and biotrauma (injury caused by inflammatory mediators released in response to mechanical ventilation), are important factors that contribute to VILI.

In addition to these factors, mechanical power (the amount of energy transferred from the mechanical ventilator to the lungs per unit of time) and lung deflation injury may also play a significant role in the development of VILI. In the following section, we discuss each of these factors in detail.

- Barotrauma

Barotrauma is a type of lung injury caused by exposure of the lung and airway to excessive positive pressure during mechanical ventilation. High peak airway pressure (Paw) can cause damage to the lung within a few minutes [37].

Webb and Tierney were among the first investigators to demonstrate barotrauma in an experimental model. Their study on anesthetized rats demonstrated that high peak inspiratory pressure caused worse lung injury with alveolar and perivascular edema [38]. Other studies have also shown the effect of high Paw on vascular permeability, alveolar cellular dysfunction, and lung mechanics [39]. 

Clinicians have long debated which pressure setting used in mechanical ventilation is most important in determining lung injury. Petersen and Baier found that barotrauma was directly proportional to the levels of peak end expiratory pressure (PEEP) and peak inspiratory pressure (PIP) [40]. The degree of regional lung distention is more critical than the absolute pressure reached [40], and it may not always be proportional to the Paw. It has been widely agreed that “Transpulmonary airway (alveolar-pleural) pressure” rather than absolute airway pressure is more important in determining lung injury [41]. This is supported by the case of trumpet players, who can reach high airway and alveolar pressures (up to 150 cm H_2_O) hundreds of times per day without developing barotrauma, as they have elevated pleural pressures resulting in lower transpulmonary pressure [41].

- Volutrauma

Volutrauma is a type of lung injury caused by overdistension of the alveoli from excessive tidal volume during mechanical ventilation. Multiple studies have shown that even short periods of exposure to excessive tidal volume can harm the lungs and compromise the effect of subsequent surfactant treatment [42,43]. The most critical factor in determining lung injury appears to be lung volume at the end of inspiration, and the injury is more pronounced if the sum of functional residual capacity (FRC) and tidal volume exceeds the total lung capacity (TLC) [5].

Studies conducted on animals and humans have demonstrated the detrimental effects of excessive tidal volume. For instance, surfactant-deficient preterm lambs that were ventilated with higher tidal volumes had lower compliances, lower ventilatory efficiencies, and lower recovery of surfactant in alveolar lavages, consistent with increased lung damage [42]. In another experimental model, Frank and colleagues demonstrated attenuation in lung injury by reducing the delivered tidal volume [44]. Similarly, in adult humans on mechanical ventilation because of acute respiratory distress syndrome, reducing the tidal volume has been shown to significantly reduce the mortality rate [45]. Limiting excessive tidal volumes through volume-targeted ventilation has also been shown to reduce pulmonary inflammation in preterm infants with respiratory distress syndrome [46].

- Which is more important in causing lung injury: barotrauma or volutrauma?

The relative importance of barotrauma and volutrauma in determining the severity of lung injury during mechanical ventilation remains a subject of debate. Both mechanisms can contribute to lung injury, and the extent to which they contribute may depend on the specific ventilatory strategy employed and the underlying pathophysiology of the lung.

Several researchers have tried to find an answer to this puzzling question. In their classic experiment, Dreyfuss and colleagues compared the consequences of normal tidal volume ventilation in mechanically ventilated rats at a high airway pressure with those of high tidal volume ventilation at a high or low airway pressure. They found that high tidal volume ventilation caused more severe lung injury than high airway pressure ventilation, even when the airway pressure was low [47]. Another study by Hernandez and colleagues found that limiting the inspiratory volume prevented an increase in the microvascular permeability, even at a high airway pressures [48]. Similarly, Carlton and colleagues found that over-inflation but not high airway pressure alone caused lung injury in newborn lambs [49]. A meta-analysis of various clinical trials in human neonates also showed that volume-targeted ventilation (VTV) is more beneficial than pressure-limited ventilation (PLV) for reducing lung and brain injury [50].

Based on these findings, it appears that volutrauma is a more critical factor in determining lung injury compared to barotrauma.

- Atelectrauma

Atelectrauma refers to a type of lung injury resulting from the cyclic opening and collapse of atelectatic but recruitable lung units- also known as RACE (repeated alveolar collapse and expansion) [51,52]. This injury occurs because of high shear forces.

Several mechanisms can lead to the development of atelectrauma, such as barotrauma resulting from higher stretching forces at the margins between open and collapsed regions of lung parenchyma [53]. Atelectasis also causes regional volutrauma due to the heterogeneous distribution of tidal volumes [54], and surfactant dysfunction could contribute to the propagation of lung injury [52,55]. The opening of the airway in an atelectatic lung also requires higher forces, leading to shear stresses that can cause epithelial disruption [41].

Studies conducted in animal models have shown the significance of PEEP in preventing atelectrauma. Dreyfuss and colleagues were one of the first groups of scientists who demonstrated the protective effect of PEEP. Their classical experiments showed that the addition of PEEP reduced lung edema and preserved the normal ultrastructural aspect of the alveolar epithelium in mechanically ventilated rats [47]. The protective effects of PEEP in preventing atelectasis and lung injury have been demonstrated by other authors as well [56]. Additionally, ventilation at lung volumes below the inflection point has been shown to cause a significant decrease in lung compliance and the progression of lung injury. Therefore, end-expiratory lung volume is a crucial determinant of the degree of lung injury during positive-pressure ventilation [57].

- Oxygen toxicity

Oxygen is commonly used in the treatment of pulmonary conditions in neonates, but its use can also result in negative effects on the lungs. In fact, the development of BPD was first described as a consequence of oxygen toxicity by Northway et al. [2] in their original description. The harmful effects of hyperoxia are mediated by reactive oxidant species (ROS), which can cause direct injury to the lungs, as well as inflammation [58]. The imbalance between pro-oxidant and antioxidant states, leading to oxidative stress, is thought to be a significant factor in the development of BPD.

There are several potential reasons why premature infants may be more susceptible to oxygen toxicity, such as underdeveloped antioxidant mechanisms [59], decreased ability to sequester hyperoxia-induced ROS generation [60], and reduced lung resident and circulating progenitor cells [61]. Hyperoxia can also lead to an influx of inflammatory cells, including neutrophils and macrophages [14]. ROS have the ability to damage membrane lipids, cellular proteins, enzymes, and nucleic acids, which can cause tissue damage and cell death [62]. This ultimately disrupts lung development by interfering with extracellular matrix assembly, cell proliferation, and vasculogenesis [14].

Several studies on both mature and immature lungs have demonstrated the harmful effects of oxygen exposure. Randomized controlled trials that compared higher versus lower oxygen concentrations for delivery room resuscitation have shown that infants receiving a higher oxygen load have increased oxidative stress and inflammation, resulting in a higher risk of developing BPD and death [63]. However, clinical data are inconclusive regarding the role of limiting oxygen exposure in preventing BPD [64].

- Biotrauma

During VILI, the accumulation of inflammatory cells and cytokines in the lungs increases [65]. This occurs because injurious ventilation strategies can cause necrosis of the plasma membrane, resulting in the release of preformed inflammatory agents and mediators that stimulate intact cells to produce similar mediators. The levels of various inflammatory cytokines, such as interleukin (IL)-1 beta (IL-1β), IL-6, IL-1 receptor agonist, tumor necrosis factor alpha (TNF-α), IL-10, macrophage inflammatory protein-2 (MIP-2), and interferon gamma (IFN-γ), are elevated in both bronchoalveolar lavage and plasma after injurious ventilation [66,67]. These pro-inflammatory mediators can lead to excessive immune system activation [68], and this inflammatory response may not be limited to the lungs. This can lead to systemic inflammatory response syndrome and multi-organ failure [69].

- Mechanical power, Stress, and Strain

Mechanical power is the product of the total inflation energy per cycle and the cycling frequency (Jules/cycle × cycles/min). It represents the amount of energy transferred from the mechanical ventilator to the lungs per unit of time.

The value of mechanical power is determined by several factors, including tidal volume, PEEP, and respiratory rate [70]. The risk of lung damage is directly proportional to the mechanical power at any given time [70]. Excessive mechanical power can disrupt the chemical bonds of the polymers composing the extracellular matrix, increasing the risk of VILI [71]. The risk of VILI at any given mechanical power depends on the lung size and the underlying lung disease. If the lungs are small or have varying mechanical properties due to heterogeneous lung disease, the risk of VILI is significantly higher at any given mechanical power [71].

Lung stress is defined as force per unit area applied directly to the lung, whereas lung strain is defined as the change in lung volume caused by lung stress [5]. Mechanical power is identified as the primary determinant of these factors [70]. Stress (i.e., transpulmonary pressure (Ptm)) coupled with strain determines lung damage [72]. The relationship between stress and strain is linearly dependent under physiological conditions, with stress being equal to the specific lung elastance (K) multiplied by strain. The specific lung elastance is the transpulmonary pressure appearing when tidal volume equals FRC. The distribution of stress and strain is crucial for determining regional lung damage [73], which is particularly relevant in patients with heterogeneous lung disease. In these patients, stress and strain are concentrated at the margins between atelectatic (collapsed) and aerated (open) lung units, which increases the risk of VILI [74].

Mechanical ventilation is a cyclic process that involves the inflation of the lungs by the tidal volume, which causes dynamic strain. PEEP is used to keep the lungs tonically inflated above their FRC, which causes additional static strain [75]. The relative contribution of dynamic and static strain to the development of VILI is a topic of debate. While some authors have suggested that large static strains may be less harmful than large dynamic strains, this remains controversial [75].

- Lung deflation injury

Lung deflation injury is a newly proposed mechanism of VILI that results from the abrupt removal of PEEP after sustained inflation. This can lead to increased lung edema and inflammation, resulting in poor lung function. The underlying pathology may be acute left ventricular decompensation, which elevates pulmonary vascular pressures at deflation, causing damage to the endothelium and resulting in alveolar edema [76].

- Pre-existing lung disease

Pre-existing heterogeneous lung disease predisposes the lung to injury during mechanical ventilation. Asymmetric lung structure with aerated, atelectatic, and consolidated lung areas causes the preferential distribution of the administered tidal volume along the path of least resistance resulting in over-distention of the aerated or good regions of the lung rather than the consolidated regions. This results in volutrauma of the relatively health lung in addition to the atelectrauma in the abnormal lung regions [54,71].

#### 3.2.2. Patent Ductus Arteriosus (PDA)

Increased blood flow to the pulmonary circulation across the PDA after birth can cause pulmonary edema and prolonged ventilatory support, which can increase the risk of VILI [77]. Excessive pulmonary blood flow, which causes neutrophil margination and subsequent inflammation, is a well-known risk factor for BPD [78]. Therefore, logically, closing the PDA should reduce the occurrence of BPD. This hypothesis is supported by some animal studies which [79] have shown an improvement in pulmonary mechanics, decreased total lung water, and a decrease in the detrimental effects of preterm birth on alveolarization with the closure of PDA with ibuprofen. However, other studies have shown that the closure of PDA does not lead to a decrease in the incidence of BPD [80]. In fact, surgical ligation of PDA has been identified as an independent risk factor for the development of BPD [81]. This suggests that the role of PDA in the pathogenesis of BPD may be more complex than previously thought.

Sellmer et al. conducted a study to investigate the effect of PDA on various neonatal morbidities. The study found that in infants less than 28 weeks gestation, the presence of a large PDA increased the odds of developing BPD by three-fold [82]. A recent study by Clyman et al. found that moderate to large PDA exposure was associated with an increased incidence of BPD in infants who required intubation for ten days or more. On the other hand, infants who received early routine treatment of PDA had lower rates of BPD. The study also found no significant difference in the rates of BPD in infants who required mechanical ventilation for less than ten days [83]. Similarly, a study conducted by Mirza et al. found a positive correlation between the duration of exposure to hemodynamically significant PDA and the incidence of BPD [84]. In contrast to the previous studies, a recent randomized controlled trial from the Netherlands investigated the effect of early treatment with ibuprofen on the incidence of BPD in extremely preterm infants with large PDA, compared to expectant management. Surprisingly, the study found that the incidence of moderate-to-severe BPD was significantly lower in the expectant management group compared to the early ibuprofen group. The absolute risk difference between the two groups was −17.6 percentage points, with a 95% confidence interval ranging from −30.2 to −5.0 [85]. This higher risk of moderate-to-severe BPD in the intervention group may be related to a decrease in vascular growth factors in preterm infants after exposure to ibuprofen [86].

Given these conflicting findings, it is clear that the relationship among the presence of PDA, its management, and the incidence of BPD is complex and likely influenced by a range of factors. Further studies are needed to better define the optimal management of PDA in preterm infants and to identify subpopulations of patients who may benefit from PDA closure.

#### 3.2.3. Sepsis

Inflammation is a critical component in the pathogenesis of BPD. Ambalavanan et al. [87] reported that higher levels of certain cytokines (IL-1β, IL-6, IL-18, IL-10, and IFN-γ) were associated with an increased risk of BPD or death. In animal models, it has been shown that infection can trigger a pro-inflammatory response leading to an interruption in normal lung development, resulting in alveolar simplification and disruption of normal angiogenesis. This produces a phenotype similar to the new BPD [88]. Inflammation can also cause dysfunction in lung endothelium, leading to structural changes in vascular development. This results in decreased density and the number of pulmonary vessels during alveologenesis [89].

In neonatal mice, inflammation has been shown to impede the expected antioxidant enzyme response to oxygen, leading to severe pulmonary vascular remodeling and aggravated alveolar remodeling [90]. Additionally, clinical studies have found a correlation between sepsis and BPD [91]. One reason for the association between sepsis and BPD in neonates could be the prolonged or higher exposure to ventilatory support and oxygen that is often required in neonatal sepsis. In contrast, some studies have shown chorioamnionitis to have a protective effect against BPD [92].

#### 3.2.4. Dysbiosis

The term “dysbiosis” refers to an altered state of microbial balance where there is a disruption in the normal community structure, leading to a disease state [93]. A growing body of evidence implicates dysbiosis in the pathogenesis of BPD [94]. During the early stages of life, the microbial community in the lungs is established and develops with the influence of various factors, including antibiotic exposure before and after birth, the use of invasive ventilation, episodes of sepsis, and inflammation [95].

In a meta-analysis of six studies examining changes in the lung microbiomes of extremely low birth weight (ELBW) infants, relative changes in the abundance of Proteobacteria, Firmicutes, and Lactobacilli were observed in the endotracheal aspirates of ELBW infants who later developed BPD [94]. Other studies have variously shown the preponderance of certain bacteria such as Ureaplasma [96], Staphylococcus, and Klebsiella species [97] and Corynebacterium [98] in infants with BPD. While there is variability among studies regarding which bacteria are associated with BPD, the common thread among these studies shows a loss of diversity and stability in the lung microbiota of infants with BPD. It is possible that the dysbiosis observed in the lung microbiota of infants with BPD may cause the development of the disease in a manner similar to the way dysbiosis in the gut can lead to necrotizing enterocolitis (NEC). It is also possible that the impact of hyperoxia on lung microbiome composition may lead to an increase in the abundance of oxygen-tolerant bacteria, which can promote pathogenic invasion, inflammation, and, ultimately, BPD. Given that the development of a normally functioning immune system is dependent, in part, on a normal microbiome [99], the dysbiosis seen in ELBW infants could contribute to an aberrant immune response from a dysfunctional immune system leading to inflammation.

Studies have also looked into the “gut–lung axis”, which refers to the impact of local microbiota on immunity at distant sites, specifically the effect of gastrointestinal (GI) tract microbiota on the immune function of the respiratory tract′s endothelial lining. Animal studies have shown an increased risk of Streptococcus pneumonia in the presence of gut dysbiosis, indicating a potential link between gut microbiota and respiratory infections [100]. Studies have shown that early and prolonged exposure to antibiotics was associated with an increased risk of BPD in very low birth weight infants [101]. This increased risk could be due to a change in gut permeability caused by antibiotic-induced gut dysbiosis [102], leading to the direct translocation of bacteria to the lungs. Alternatively, gut dysbiosis could generate an abnormal inflammatory response due to the production of metabolites, such as sphingolipid and polysaccharide A [103].

Over the past decade, there has been an increasing focus on volatile organic compounds (VOCs) and their potential involvement in the development of BPD. VOCs are gaseous byproducts of metabolic processes in cells, which could potentially contribute to BPD by promoting inflammation [104] and exacerbating oxidative stress [105]. They may prove to be useful early clinical biomarkers for BPD [106].

#### 3.2.5. Nutrition

It has been widely observed that poor postnatal growth is linked to an increased risk of developing BPD [107]. Prenatal and postnatal growth restriction has been identified as significant risk factors for the development of BPD [108,109]. A study that compared the nutrition and growth of preterm infants who developed BPD to those who did not found that the former group received a significantly lower calorie-to-protein ratio than the latter [109]. This may be attributed to the higher severity of illness in these infants, which could limit their ability to receive adequate nutrition of both sufficient volume and quality. They further found that at term-corrected age, infants with BPD had significantly lower head circumference and length z-scores compared to infants who did not, while their weight z-scores were similar. Although weight is the primary tool used to assess postnatal growth, adequate lung development may be closely linked to linear growth [109]. Thus, it is important to evaluate proportionate growth during the neonatal intensive care unit (NICU) course. In addition, some evidence suggests that excessive intake of fluids and sodium during the early postnatal period may also contribute to the development of BPD [91]. It is theorized that higher fluid intake in the first postnatal week impairs the physiological contraction of extracellular water, which could lead to a greater need for respiratory support. This may be because of worsened pulmonary edema and an increased risk of a hemodynamically significant PDA [110].

Studies have also looked into the potential benefits of vitamin supplementation for supporting lung growth, with a particular focus on vitamin A. This vitamin has been shown to play a role in alveolar development and maintenance [111], and ELBW infants are often deficient in vitamin A because of limited placental transfer in the third trimester [112]. Some research suggests that intramuscular vitamin A injections may confer a slight benefit in reducing oxygen dependency at 36 weeks postmenstrual age for very preterm infants [113]. However, vitamin A supplementation has not been adopted as a standard of care across NICU because of the need for multiple injections and only a marginal benefit. A recent study [114] showed no decrease in the risk of BPD with oral vitamin A supplementation.

Vitamin D deficiency has been linked to impaired alveolar development, inhibition of type II alveolar cells and fibroblasts, and reduced surfactant production and antioxidant effect in animals [115]. Antenatal vitamin D deficiency was also found to be associated with increased airway smooth muscle mass and airway collagen synthesis, as well as elevated bronchoalveolar lavage neutrophil counts and depressed lymphocyte counts in animal models [116]. A recent small, randomized control trial [117] reported that preterm infants who received early vitamin D supplementation had a lower risk of developing BPD; however, another recent trial [118] found that although there was a trend towards decreased BPD with vitamin D supplementation, it did not reach statistical significance. Further studies are required to explore the association between vitamin D supplementation and BPD.

## 4. Conclusions

To summarize, the pathogenesis of BPD is multifactorial, with risk factors including preterm birth, mechanical ventilation, oxygen toxicity, infection, and genetic factors. The lung injury caused by BPD is believed to result from the interplay between lung immaturity, fetal and neonatal exposure to inflammatory mediators, oxidative stress, and abnormal growth factor signaling. The resulting lung injury leads to impaired alveolar and vascular growth, resulting in a characteristic pattern of abnormal lung development, including decreased alveolarization, thickened interstitium, and disrupted vasculature. The pathogenesis of BPD is still not fully understood, but advances in research may help to identify potential therapeutic targets for this challenging condition.

## Data Availability

Not applicable.
